# Phytochemistry of *Verbascum* Species Growing in Iraqi Kurdistan and Bioactive Iridoids from the Flowers of *Verbascum calvum*

**DOI:** 10.3390/plants9091066

**Published:** 2020-08-20

**Authors:** Hawraz Ibrahim M. Amin, Faiq H. S. Hussain, Gianluca Gilardoni, Zaw Min Thu, Marco Clericuzio, Giovanni Vidari

**Affiliations:** 1 Department of Chemistry, College of Science, Salahaddin University-Erbil, Erbil 44001, Iraq; 2 Paitaxt Technical Institute-Private, Erbil 44001, Iraq; 3 Department of Medical Analysis, Faculty of Science, Tishk International University, Erbil 44001, Iraq; faiq.hussain@ishikuniversity.onmicrosoft.com; 4 Departamento de Química y Ciencias Exactas, Universidad Técnica Particular de Loja, Calle Marcelino Champagnat s/n, Loja 110107, Ecuador; gianluca.gilardoni@gmail.com; 5 Department of Chemistry, Kalay University, Kalay 03044, Myanmar; zawminthu87@gmail.com; 6 Department of Sciences and Technological Innovation (DISIT), Università del Piemonte Orientale, 15121 Alessandria, Italy; marco.clericuzio@uniupo.it; 7 Dipartimento di Chimica, Università di Pavia, Via Taramelli 12, 27100 Pavia, Italy

**Keywords:** phenolics, terpenoids, iridoid, ajugol, aucubin, *Verbascum*, antiradical activity, Kurdish traditional medicine

## Abstract

Traditional medicine is still widely practiced in Iraqi Kurdistan, especially by people living in villages on mountainous regions; medicinal plants are also sold in the markets of the large towns, such as at Erbil, the capital of the Kurdistan Autonomous Region. About a dozen of *Verbascum* species (Scrophulariaceae) are commonly employed in the Kurdish traditional medicine, especially for treating burns and other skin diseases. However, the isolation of bioactive secondary metabolites from these plants has not been the subject of intense scientific investigations in Iraq. Therefore, the information reported in the literature about the species growing in Kurdistan has been summarized in the first part of this paper, although investigations have been performed on vegetable samples collected in neighbouring countries, such as Turkey and Iran. In the second part of the work, we have investigated, for the first time, the contents of a methanol and a hydromethanol extract of *V. calvum* flowers. The extracts exhibited weak antimicrobial activities, whereas the methanol extract showed significant antiproliferative effects against an A549 lung cancer cell line. Moreover, both extracts exhibited a significant dose-dependent free radical scavenging action against the 2,2-diphenyl-1-picrylhydrazyl (DPPH) radical, comparable to that of ascorbic acid. In the subsequent phytochemical study, a high phenolic content was determined in both extracts by the Folin–Ciocalteu assay and medium-pressure liquid chromatographic (MPLC) separation led to the isolation of iridoid glucosides ajugol and aucubin from the methanol extract. In conclusion, the high anti-inflammatory effects of aucubin and the remarkable antioxidant (antiradical) properties of the extracts give scientific support to the traditional use of *V. calvum* flowers for the preparation in Kurdistan of remedies to cure skin burns and inflammations.

## 1. Introduction

The majority of Kurds inhabit a mostly mountainous land called Kurdistan that includes adjacent parts of Iran, Iraq, Syria and Turkey. The name of Kurdistan means the ‘land of the Kurds’ because it is the traditional country where the Kurdish people have been constituted ethnically as a homogeneous community since the dawn of recorded history, and where they have developed their culture and shaped their destiny. Indeed, their diverse and distinctive social and traditional lifestyle has been well preserved until today. However, although Kurds form a nation, they do not have their own state. About 4.2 million Kurds constitute approximately 19% of Iraq’s overall population and live in northern Iraq, in the Kurdistan Autonomous Region, which has received limited administrative autonomy from the Baghdad central government in 1991. The region comprises the governorates of Dohuk, Erbil, Halabja and Sulaymaniyah.

**Figure 1:**
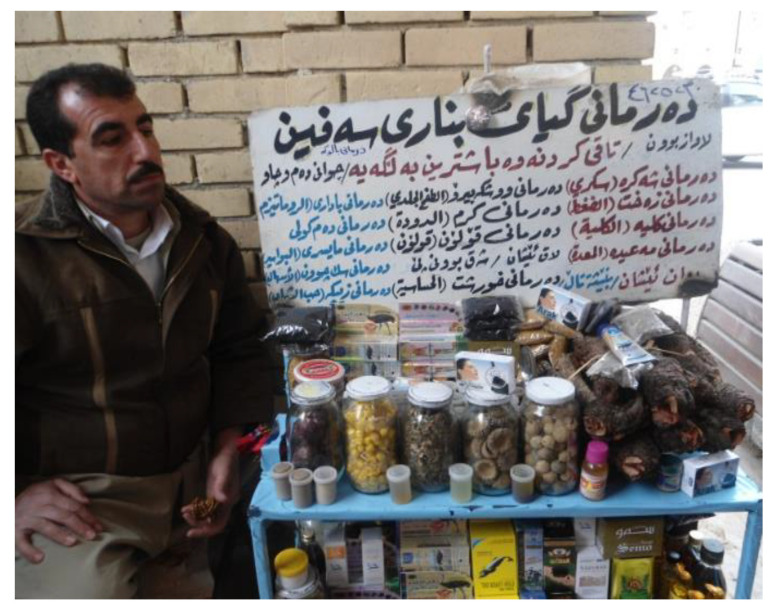
Kurdish seller of traditional remedies (Photo taken by H.I.M.A.).

A Neanderthal burial discovered at Shanidar cave (number IV in the series of skeletons) in northern Iraq has proved to be of singular importance. In fact, in the soil samples taken around the skeleton, dated approximately 60,000 years ago, the pollens of eight different flowers were collected. They belong to medicinal plants still growing in Iraqi Kurdistan [1]. This finding is thus a clear piece of evidence that herbal medicine is probably an ancient practice in the mountains and plains of Kurdistan. Actually, a well-organized form of medicine, which made intense uses of plant-derived drugs, remedies, potions and oils, can be traced back in Iraq and Kurdistan to the Sumerian period (3000–1970 B.C.) and then to the Babylonian and Assyrian periods (1970–539 B.C). Later on, this knowledge was translated and enriched by the Arab physicians during the Abbasid period (500–1038 A.D) and survived until modern times. The Second Gulf War in 1991 and the subsequent economic sanctions caused an acute shortage of modern medicines in the country, resulting in an increased use of herbal remedies. In fact, more than 130 plants are still used today in Kurdistan, especially in villages and rural areas (Figure 1) [2,3]. The Qaysari bazaar, located in the centre of Erbil, the capital of the Kurdistan Autonomous Region, has 21 herbalist shops trading natural medicinal products [4]. Thirty-two plants belonging to 23 families are used by traditional healers in the Erbil region to treat various diseases [5], whereas sixty-six plants, belonging to sixty-three genera within thirty-four plant families, are used for treating ninety-nine different types of ailments and diseases in the Province of Sulaymaniyah [6].

Herbs used for curing gastro-intestinal disorders clearly predominate. This finding can be due to the abundance of these diseases among the Kurdish people, and/or to the broad applicability of these terms, as they can also include reduction of cholesterol level, abdominal pain, flatulence, colic, etc. Other diseases cured by using plants are related to the circulatory and respiratory systems, the urogenital and the metabolic systems, including diabetes, various types of inflammation and infection, skin burns, and liver problems [4,5,6]. 

Despite the wide use of herbal remedies, phytochemical studies on Kurdistan medicinal plants are still in their infancy and only a few papers have been published so far that describe the structures and bioactivities of isolated metabolites [2,3,7,8,9,10,11,12,13]. In continuation of our researches on Kurdistan medicinal plants, we direct our attention toward the genus *Verbascum* (mullein). This large genus of the family Scrophulariaceae has about 130 accepted names in *The Plant List* database, while about 85 are synonyms and the others are unresolved names [14]. Several *Verbascum* species have been reported to exhibit antiseptic, astringent, demulcent, emollient, expectorant, sedative, diuretic, antimalarial properties. Included in different traditional medicines, these plants are used for treating wounds, haemorrhoids, chilblains, respiratory ailments, inflammations, migraine, arthritic problems, acne, eczema and other types of inflammatory skin conditions [15].

Numerous *Verbascum* species grow wild in various ecosystems existing in territories extending from Aegean Islands to Anatolia and Iran. A total of twenty-seven species can be found in Iraq, mainly on high-mountain regions [16,17], whereas about fifteen are reported to grow in Kurdistan. They include *V. agrimoniifolium* Huber-Morath (accepted name), *V. alceoides* Boiss. & Hausskn. (unresolved name), *V. assurense* Bornm. & Hand.-Mazz. (unresolved), *V. calvum* Boiss. & Kotschy (unresolved), *V. carduchorum* Bornm. (unresolved), *V. cheiranthifolium* Boiss. (unresolved), *V. froedinii* Murb. (unresolved), *V. geminiflorum* Hochst. (unresolved), *V. laetum* Boiss. & Hausskn. (unresolved), *V. macrocarpum* Boiss. (accepted), *V. oreophilum* C. Koch (accepted), *V. ponticum* Stef. (unresolved), *V. pseudodigitalis* Nábělek (unresolved), *V. songaricum* Schrenk (accepted), *V. speciosum* Schrad. (accepted), and *V. thapsus* L. (accepted). Most of them are used to treat skin burn inflammations and mycodermatitis [2].

Only a limited number of these species have been subjected to phytochemical studies. They have been carried out outside Kurdistan, mostly by research groups working in Turkey, Iran, and India, where the same plants are commonly used in the local traditional medicine. The results of these investigations have been summarized in the first part of this inaugural paper on secondary metabolites from *Verbascum* plants occurring in Kurdistan. The literature reported in Reaxys and Google Scholar databases until April 2020 has been reviewed. In the second part of this paper, we describe the preliminary results of our study on the content of a methanol extract of *V. calvum* flowers.

## 2. Literature Data about the *Verbascum* Species Growing in Kurdistan

**Figure 2:**
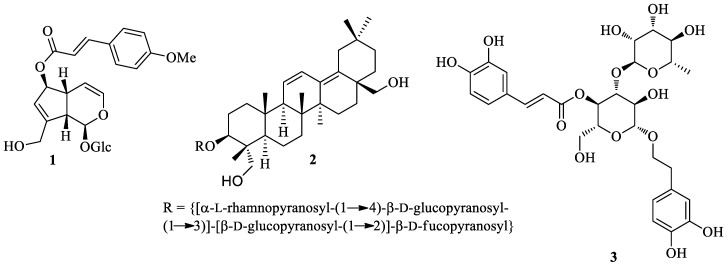
Structures of 6-*O*-[(*E*)-*p*-methoxycinnamoyl]aucubin (**1**). songarosaponin A (**2**), and verbascoside (**3**).

**Figure 3:**
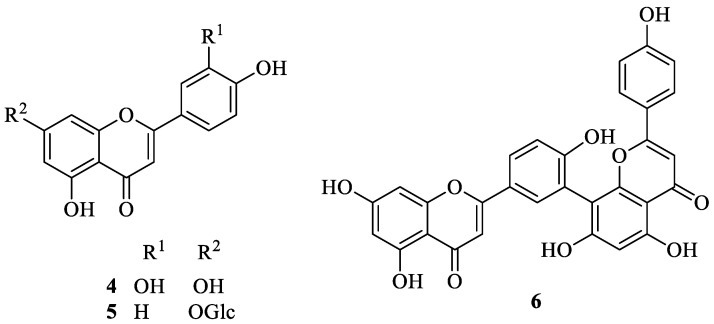
Characteristic flavonoids isolated from *Verbascum thapsus*.

**Figure 4:**
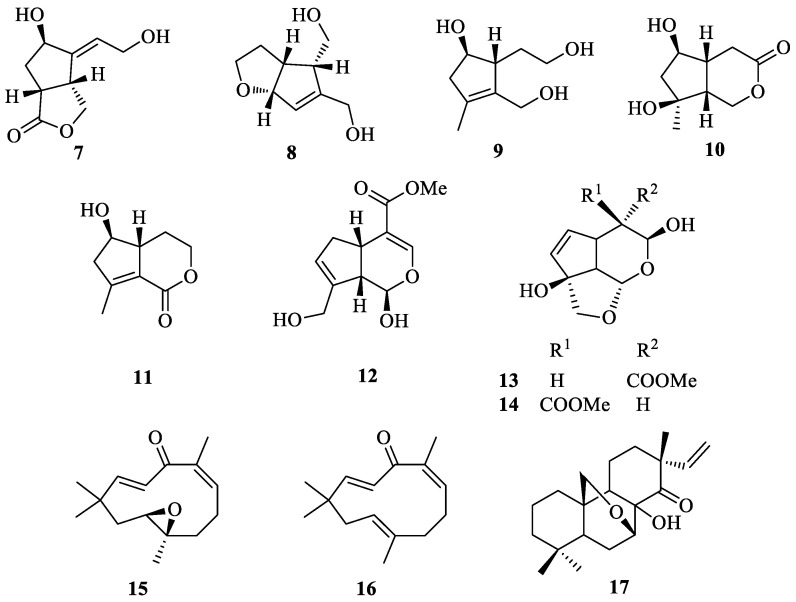
Characteristic iridoids and other terpenes isolated from *Verbascum thapsus*.

**Figure 5:**
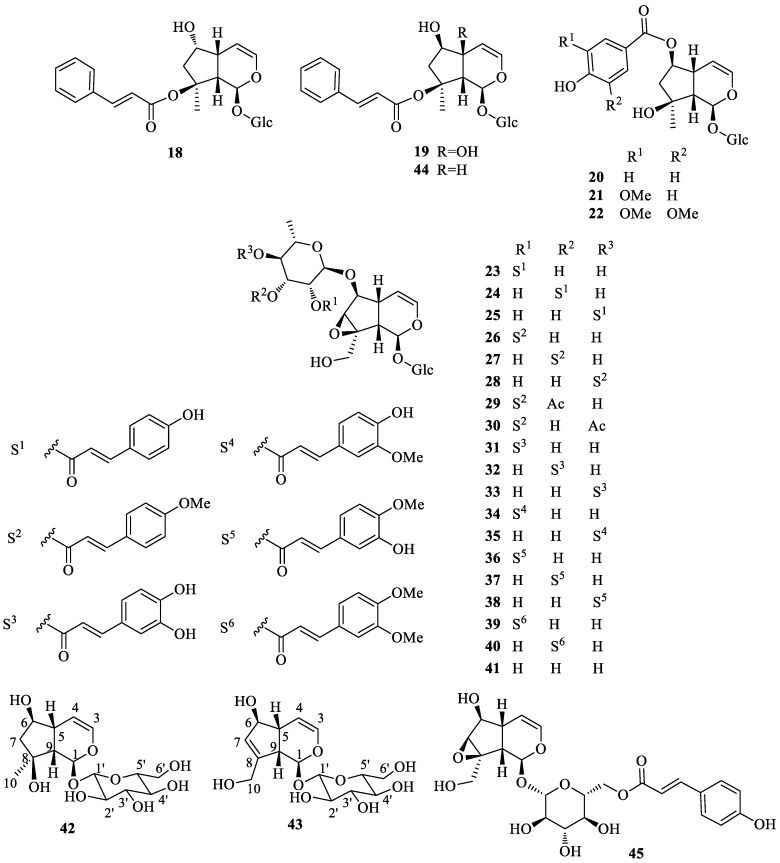
Characteristic iridoid glycosides isolated from *Verbascum thapsus*.

**Figure 6:**
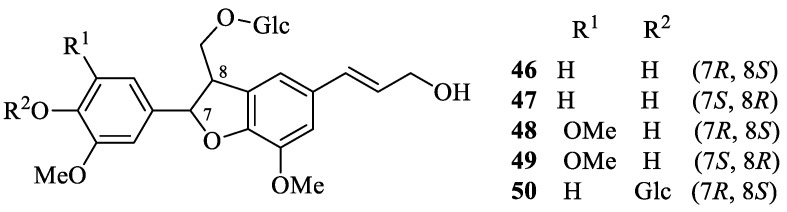
Characteristic lignan glycosides isolated from *Verbascum thapsus*.

**Figure 7:**
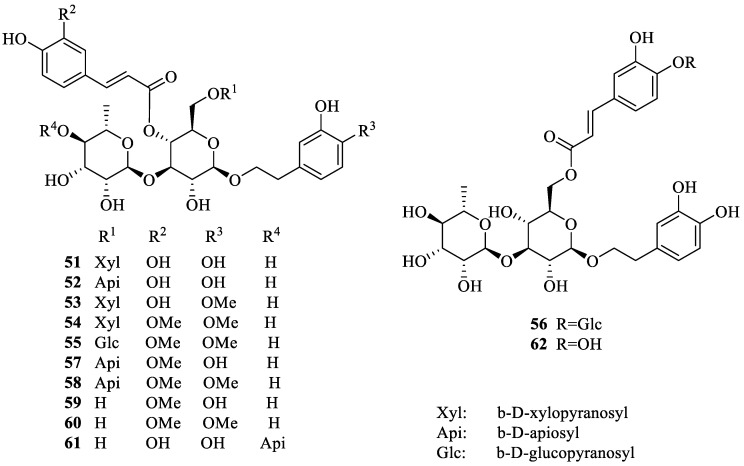
Characteristic phenylethanoid glycosides isolated from *Verbascum thapsus*.

**Figure 8:**
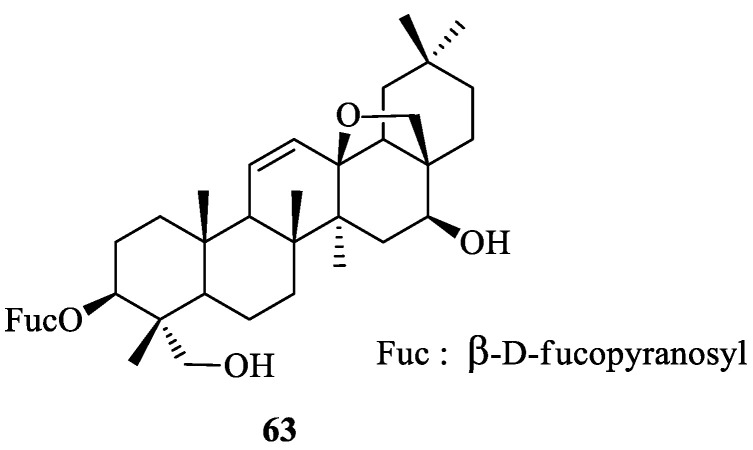
Saponine 3-*O*-fucopyranosylsaikogenin F from *Verbascum thapsus*.

Many natural products, belonging to different biosynthetic pathways (terpenoids, shikimic acid derivatives and mixed metabolites), have been described in the only four *Verbascum* species growing in Kurdistan that have been investigated so far: *Verbascum cheiranthifolium* Boiss., *Verbascum songaricum* Schrenk, *Verbascum speciosum* Schrad. and *Verbascum thapsus* L. These results, together with the plant traditional uses and the known biological activities, are listed in Table 1. Their molecular structures are represented in Figure 2, Figure 3, Figure 4, Figure 5, Figure 6, Figure 7 and Figure 8.

## 3. Phytochemical Studies on *Vebascum Calvum*

**Figure 9:**
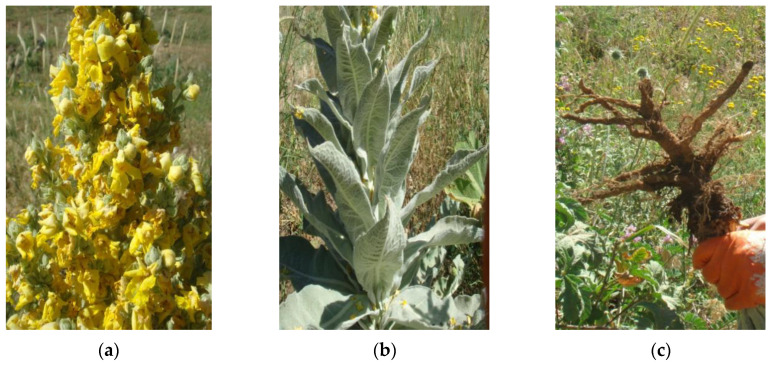
Flowers (**a**), leaves (**b**) and roots (**c**) of *Verbascum calvum* (Photos taken by H.I.M.A.).

*Verbascum calvum* Boiss. & Kotschy (Figure 9) is used in the folkloric medicine of Iraqi Kurdistan for the treatment of burns, especially skin burns and other skin inflammations.

**Figure 10:**
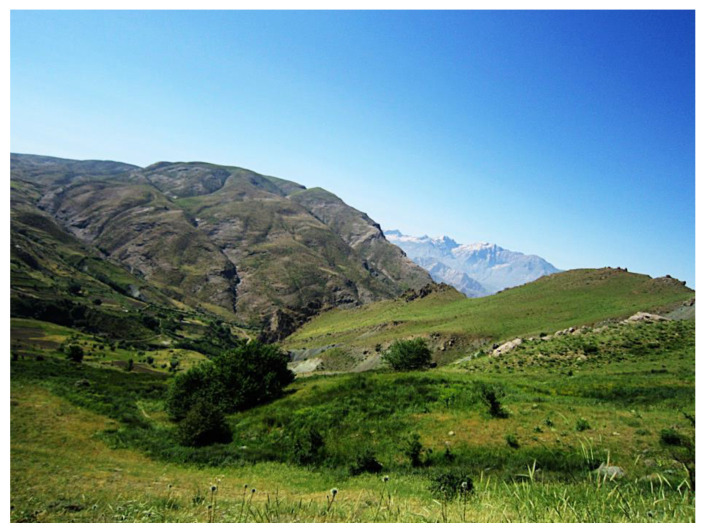
Halgurd mountain where *V. calvum* was collected (Photo taken by H.I.M.A.).

For treating skin burns, the plant is used in two traditional manners. During the plant growing season, fresh leaves are applied topically on cleaned burn wounds. When the plant cannot be found fresh, dried stem leaves and flowers are boiled in water for about 1 h; the liquid is then concentrated by evaporation to give a gelatinous cream that is gently smeared on the cleaned wound. In the Kurdistan region, *V. calvum* is found on Halgurd mountain (Rawanduz) in the Erbil province (Figure 10), at Kany Mase (Zakho) and in the Sulaimaniya district [16,17].

Neither phytochemical investigations nor an evaluation of biological activities has been carried out so far on extracts of *V. calvum*. Therefore, in our ongoing investigations on Kurdish medicinal plants [2,3], we decided to examine the phytochemical contents and the biological activities of polar extracts of the flowers, since an aqueous infusion is usually employed for the preparation for folkloric remedies.

### 3.1. General Experimental Techniques and Procedures

For most general experimental techniques and procedures, see reference [80]; ^1^H NMR and ^13^C NMR chemical shifts are relative to signals of residual C*H*D_2_OD and ^12^*C*D_3_OD in MeOH-*d*_4_ (δ_H_ 3.27 (central line of a quintuplet) and δ_C_ 49.0 (central line of a septuplet), respectively). Preparative medium-pressure liquid chromatographic (MPLC) separations were carried out on a Biotage Isolera instrument.

### 3.2. Plant Material

Flowers, leaves, and roots of *V. calvum* Boiss & Kotschy (Figure 9) were separately collected on Halgurd mountain (Figure 10) on June 19th, 2011; GPS position: 36°40′30.40”N, 44°50′7.40”E. The plant was identified by botanists A. H. Al-khayyat and Abdullah S. A. from the University of Salahaddin-Hawler/Iraq. A voucher specimen (No. VC 6823) has been deposited at the Salahaddin University herbarium (ESUH). The plant raw materials were air dried at room temperature for 10 days in a ventilated room, protected from light. After drying, the flowers were powdered by an electric mill grinder.

### 3.3. Extraction of V. Calvum Flowers

Powdered air-dried flowers of *V. calvum* (210 g) were suspended in hexane (1.25 L). The flask was immersed in an ultrasonic bath for 5 min and then left at room temperature for 1 h. Subsequently, the mixture was decanted and filtered. This process was repeated 3 times; the extracts were combined and the solvent was removed by a rotary evaporator. The resulting residue (A, 1.78 g, 0.85% *w*/*w*) was stored at 4 °C, protected from light. Subsequently, the biomass was extracted with MeOH (1 L). The mixture was immersed in an ultrasonic bath for 5 min and then left at room temperature for 1 h; subsequently, the mixture was decanted and filtered. This process was repeated 3 times, each time using a fresh amount of solvent (1 L). The extracts were then combined and the solvent was removed by evaporation under vacuum. The resulting residue (B, 35.5 g, 16.9% *w*/*w*) was stored in a vial at 4 °C, protected from light. Finally, the biomass was extracted with 800 mL of a mixture of MeOH/ H_2_O (70:30), following the procedure previously described 3 times. The extracts were then pooled together and the methanol was removed by a rotary evaporator; the residual aqueous phase was finally lyophilized to give a crude residue (C, 16.05g, 7.6% *w*/*w*) that was stored in a vial at 4 °C, protected from light.

### 3.4. Preliminary Phytochemical Analysis of Residues B and C

Residues B and C were preliminary analyzed on analytical thin-layer chromatographic (TLC) plates under two chromatographic conditions: (a) RP-18 plates, eluted with MeOH/H_2_O (1:1); (b) silica-gel 60, eluted with EtOAc/*n*-BuOH/HCO_2_H/H_2_O (5:3:1:1). After drying, the plates were inspected under UV light at 366 nm before and after exposure to NH_3_ fumes, or under visible light after spraying them with a FeCl_3_ solution (2.7 g in 100 mL of 2M HCl), or under visible and UV light after spraying with an AlCl_3_ solution (1g in 100 mL of EtOH), or with an ethanolic solution of 2,2-diphenyl-1-picrylhydrazyl (DPPH), or with 0.5% vanillin in sulfuric acid/EtOH (4:1), followed by heating at 110 °C for about 1 min [80]. The colors of the spots indicated the presence of phenols, phenolic acids, flavonoids, and terpenoids. Moreover, the extracts B and C were very similar qualitatively.

### 3.5. Preliminary Chromatographic Purification of Residues B and C

To remove chlorophylls and residual highly lipophilic components, a sample of residue B (1.1 g) was filtered through an SPE C-18 cartridge (Discovery^®^ DSC-18 SPE Tube, bed wt. 10 g, volume 60 mL, Supelco) previously conditioned with MeOH (100 mL), followed by H_2_O (100 mL). The sample was dissolved by stirring in MeOH/H_2_O (90:10) in an ultrasonic bath for 1 h, loaded on the top of the column, and eluted by 200 mL of MeOH/H_2_O (90:10). The liquid was taken to dryness under vacuum. Subsequently, to remove tannins and other polymeric material, the resulting residue (1.07 g) was filtered with a flow of 4 mL/min through a Sephadex LH-20 column (50 g, Sigma) that had previously been swollen in MeOH/H_2_O (80:20) overnight. The sample was dissolved in 10 mL of MeOH/H_2_O (80:20) in an ultrasonic bath for 1 h and, after loading on the column, was eluted by equal volumes (250 mL each) of MeOH/H_2_O (80:20), MeOH/H_2_O (65:35), MeOH/H_2_O (50:50), and Me_2_CO/H_2_O (70:30) (in this order). The solutions were combined and evaporated to dryness under vacuum to give residue B’ (1.05 g). An analogous procedure was followed to remove possible lipophilic and tannin contaminants from a sample (100 mg) of extract C to give residue C’ (90 mg).

### 3.6. Folin–Ciocalteu Assay

The total phenolic contents of the extracts B’ and C’ were determined by the Folin–Ciocalteu assay, according to the method described by Singleton et al. [81]. Ten percent EtOH was the solvent for the standard and the samples. A calibration line was constructed using gallic acid (Sigma-Aldrich) as the standard in the concentration range of 1–5 µg/mL. The equation of the calibration curve was y = 0.0876(7) x + 0.006(2). Absorbances of tested samples were read at 760 nm, against a blank containing 1 mL of 10% EtOH. Analyses were conducted in triplicate. The total phenolic contents in samples B’ and C’, expressed as gallic acid equivalent/100 g extract, were 79.56 (±1.26) g and 74.91 (±0.19) g, respectively.

### 3.7. Anti-radical Activity Test

**Figure 11:**
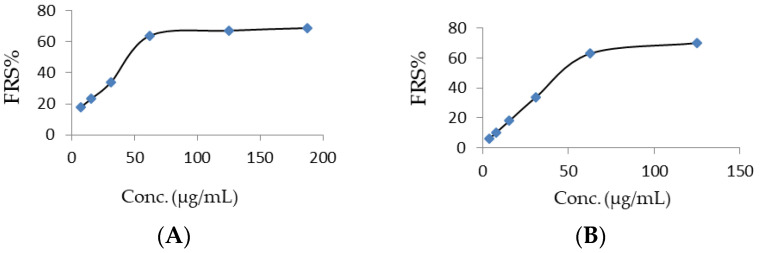
Per cent free radical scavenging (FRS%) activity vs. sample concentration (µg/mL) curves for extracts B’ (**A**) and C’ (**B**) in the 2,2-diphenyl-1-picrylhydrazyl (DPPH) test.

The free radical scavenging (FRS) activity of residues B’ and C’ was’ determined using the 2,2-diphenyl-1-picrylhydrazyl stable radical (DPPH, Sigma-Aldrich) method [82]. B’ and C’ were separately dissolved in MeOH at a concentration of 10 mg/mL. Stock solutions were then serially diluted with MeOH, giving test solutions at the final concentrations of 7.6, 5.0, 2.5, 1.25, 0.6, and 0.3 mg/mL for B’ and 5.0, 2.5, 1.25, 0.6, 0.3, and 0.15 mg/mL for C’. Three solutions of ascorbic acid (Sigma-Aldrich), used as the reference standard, were prepared with concentrations of 10, 5.0, and 2.5 mg/mL in MeOH. Test mixtures are prepared by adding 100 µL of each extract (or standard) solution to 3.9 mL of DPPH solution, freshly prepared dissolving DPPH in methanol/KH_2_PO_4_ and NaOH buffer (50/50 v/v) at a concentration of 6 × 10^−5^ M (pH 7.4). After 30 min of incubation at room temperature in the dark, the absorbance was measured at 515 nm using a UV-Visible spectrophotometer (Lambda 25 UV/VIS spectrometer N.3903, Perkin Elmer instruments, Massachusetts, USA). FRS activity was expressed as a percent compared with the control, that consisted of the DPPH solution (3.9 mL) and MeOH (100 µL). The percent inhibition of the DPPH radical by the tested solution was calculated using the following formula FRS% = [(A_control_ − A_ample_)/A_control_] × 100. The analyses were carried out in triplicate and the results are expressed as mean ± SD. The concentration of the sample reducing 50% DPPH (EC_50_) was determined by plotting FRS% against the sample concentration (Figure 11).

### 3.8. Antibacterial Activity Test

The antibacterial activities of B’ and C’ against bacteria *Staphylococus aureus* (ATCC 6538), *Staphylococus mutans* (ATCC 25175), *Staphylococus pyogenes* (ATCC 19615), and *Escherichia coli* (ATCC 10356) were determined as the Minimum Inhibitory Concentration (MIC) and the Minimum Bactericidal Concentration (MBC), both expressed in µg/mL. The MIC is defined as the lowest concentration of an antimicrobial that inhibits the visible growth of a microorganism after overnight incubation, whereas the MBC corresponds to the lowest concentration of an antimicrobial that results in bacterial death after sub-culture onto an antibiotic-free medium. Gentamicin was used as the positive control. Each strain was first incubated for 18–20 h in Tryptone Soya Broth (TSB; Oxoid LTD., Basingstoke, Hampshire, England) at 37 °C. The culture thus obtained was then centrifuged at 3000 rpm for 20 min to separate the cells from the culture medium. The culture medium was removed and the cells were resuspended in IsoSensitest Broth (ISB; Oxoid LTD., Basingstoke, Hampshire, England). Subsequently, the bacterial suspension was appropriately diluted to obtain an absorbance of 0.2 at 655 nm, measured by a spectrophotometer (Jasco-320 Uvidec Japan Spectroscopic Co., Ltd., Tokyo, Japan). This absorbance corresponded to a concentration of microorganisms in the range of 1 × 10^7^–1 × 10^8^ CFU /mL.

The MIC of each sample was determined by the method of dilution with culture medium [83]. ISB series of tubes containing constantly increasing concentrations of B’ or C’ were inoculated with the same amount of each microbial suspension. After 18–24 h of incubation at 37 °C, the MIC was determined by turbidity and expressed as the percentage volume of the extract used in the assay compared to the total volume (volume of microbial rich medium (mL) + volume antimicrobial agent (mL)) in the tube (% v/v). The MBC values were calculated by inoculating aliquots of culture medium taken from tubes where the MICs had been determined, in test tubes containing extract-free culture medium. Three replicates were performed for each test. The MICs of B’ and C’ against *S. aureus*, *S. mutans*, *S. pyogenes*, and *E. coli* were, respectively, >1530 and >1500, 918 and 1050, 612 and 750, >1530 and >1500 µg/mL. The MBCs of B’ and C’ were >1500 µg/mL against all the tested strains. For comparison, the values determined for gentamicin were in the range of 0.25–1 µg/mL.

### 3.9. Antifungal Activity Test

The antimycotic activity of samples B’ and C’ were tested using our collection of fungal strains, namely *Candida albicans* (Robin) Berkhout, *Microsporum canis* E. Bodin ex Guég., *Aspergillus niger* Tiegh., and *Bipolaris oryzae* (Breda de Haan) Shoemaker. Fungi were maintained on Sabouraud (SAB) agar medium (1 L of distilled water, 30 g of glucose, 10 g peptone). Suspensions were prepared from a 24-h-old culture in the case of *C. albicans* and one-week-old cultures in the case of the other strains. The antifungal activities were evaluated by means of the microdilution method in 96-well plates [84,85]. After proving that the extracts were soluble, serial dilutions of the extracts were prepared directly using the SAB medium inoculated with the tested fungus. The following concentrations were used: 5, 2.5, 1, 0.5, 0.25, 0.125, 0.06, 0.03, 0.015, 0.007, 0.003 mg/mL. A volume of 50 µL of each dilution was distributed in each well. Three replicates were performed for each test and dilution. The inoculated plates were incubated at 37 °C for *C. albicans* and at 27 °C for the other fungi. The MICs (minimum inhibition concentrations) were determined after 24 h in the case of *C. albicans* and *A. niger*; for the other microorganisms, the MICs were recorded after 5 days. The MIC values are reported in Table 2. Fluconazole (MIC = 1 µg/mL against *C. albicans*) was used as the reference antifungal compound.

### 3.10. Tumor Cell Viability Test (MTS Assay)

The MTS cell proliferation assay protocol is based on the reduction of the MTS [3-(4,5-dimethylthiazol-2-yl)-5-(3-carboxymethoxyphenyl)-2-(4-sulfophenyl)-2H-tetrazolium] yellow tetrazolium compound by NAD(P)H-dependent dehydrogenase enzymes in metabolically active cells to a purple formazan salt, that is soluble in cell culture media [86]. The formazan salt has a maximum of absorbance in the UV spectrum at 490 nm. The measure of the absorbance can be directly related to the number of viable (living) cells.

MCF-7 breast (ATCC No. HTB-22) and A549 lung cancer cells (ATCC No. CLL-185) were grown in flasks for several days in an incubator (Forma Scientific) at 37 °C under a humidified atmosphere added of 5% CO_2_, changing the liquid growth medium (RPMI 1640 (Euroclone, Europe) supplemented with 10% fetal bovine serum (Gibco, UK), and 0.005% L-glutamine, penicillin and streptomycin (Life Technologies, Milan, Italy)) whenever needed. When a cell culture reached confluency, a small amount of trypsin was added to the medium in order to separate the cells from the flask; after 3 min of incubation at 37 °C, 1 mL of FBS was added to stop the action of trypsin and avoid cells membrane degradation. The cell-containing medium was then transferred into a cell strainer and centrifuged (ALC 4232 Centrifuge) at 1000 rpm for 10 min. The resulting pellet was resuspended in the growth medium (1 mL), and the cells were separated using an automatic pipette and counted using a counting chamber and trypan blue as dye. After a proper dilution, separated cells were plated in a 96-well flat-bottom microplates (Cellstar, Greiner bio-one) at a density of 3 × 10^3^ cells in 100 µL of growth medium in each well. After 2 h of incubation, the growth medium was replaced with 100 µL of test medium (RPMI 1640 added with 0.005% L-glutamine, penicillin and streptomycin) and the microplate was left in the incubator for 24 h. Five test solutions (1 mL each) with concentrations equal to 600, 60, 6, 0.6, and 0.06 µg/mL, respectively, were prepared by diluting a stock solution of sample B’ (60 mg/mL MeOH) with the test medium. The test medium in the wells was replaced with a solution (100 µL) of increasing sample concentration. For each sample tested, five replicates were performed for each dilution. The microplate was then further incubated for 24 h; subsequently, the sample containing medium was replaced with fresh test medium (100 µL) and 20 µL of MTS tetrazolium reagent (CellTiter 96^®^ – AQueous One Solution Cell Proliferation Assay, Promega, USA). After 2 h of incubation, the absorbance was measured at 490 nm using a plate reader (BioRAD Model 550 Microplaate Reader). The IC_50_ (µM) ± S.D values of cisplatin used as the cytotoxic reference compound were 17 (±4) and 13 (±2) against MCF-7 and A549 cell lines, respectively. The control was the test medium + 1% MeOH.

The percentages of growth inhibition of tumor cells (treated with different concentration of extract B’) compared to the control are shown in Table 3. The graphical results of the antiproliferative activities of extract B’ are represented in Supplementary Material.

### 3.11. Chromatographic Separation of Residue B’

Residue B’(1g) was separated on a medium pressure liquid chromatography using a hand-packed reversed-phase column (LiChroprep RP18, 25–40 mm, 120 g, MerckMillipore). Solvents A and B of the mobile phase were H_2_O and MeOH, respectively. A linear gradient was applied from an A/B mixture (80:20) to 100% B, over 4.8 h at room temperature, at a flow rate of 10 mL/min; dual UV detection wavelengths set at 215 and 220 nm. The column was eventually washed with 100% MeOH for 3 min to elute strongly adsorbed compounds. Fifty-three fractions (8 mL each) were collected; the solvent in the tubes was evaporated using a centrifuge under vacuum and a liquid nitrogen trap, and the residues were weighed. The overall recovery of the chromatographed mixture was 95.1%. Then, the content of each fraction was analyzed by TLC on analytical silica gel 60 (GF_254_, Merck) plates, eluted with EtOAc/*n*-BuOH/HCO_2_H/H_2_O (5:3:1:1), and on RP-18 (Sigma-Aldrich) plates, eluted with MeOH/H_2_O (1:1). Spots were detected under UV light at 254 and 366 nm and by spraying the plate with 0.5% vanillin in sulfuric acid/EtOH (4:1) [80], followed by heating at 105 °C for about 1 min. Fractions 7 (42.8 mg) and 12 (10.8 mg), obtained as colorless powders, were homogeneous and were directly submitted to spectroscopic analysis. Comparison with literature data indicated their identity with aucubin (**43**) [87,88] and ajugol (**42**) [89], respectively.

### 3.12. Spectroscopic Data of Compounds **43** (Aucubin) and **42** (Ajugol)

Aucubin (**43**): UV λ_max_ (MeOH) 210 nm; IR (KBr): 3270 (OH), 2918, 1655 (C=C), 1230, 1045 cm^−1^; ^1^H NMR (300 MHz, MeOH-*d*_4_): Table 4; ^13^C NMR (75 MHz, MeOH-*d*_4_): Table 4. LC-ESIMS (positive ion mode): *m/z* 369.18 [M+Na]^+^; 714.96 [2M+Na]^+^.

Ajugol (**42**): UV λ_max_ (MeOH) 220 nm, IR (KBr): 3400 (OH), 1660 (C=C) cm^−1^; ^1^H NMR (300 MHz, MeOH-*d*_4_): Table 4; ^13^C NMR (75 MHz, MeOH-*d*_4_): Table 4. LC-ESIMS (positive ion mode): *m/z* 371.19 [M+Na]^+^; 719.19 [2M+Na]^+^.

All NMR and ESI-MS spectra of aucubin and ajugol are reported in Supplementary Material.

### 3.13. Acidic Hydrolysis of Compounds **42** and **43**

Compounds **42** and **43** (3.0 mg each) were separately dissolved in 2M HCl in a sealed vial and heated at 100 °C for 45 min. After cooling to room temperature and extraction with CHCl_3_, the aqueous layer was repeatedly evaporated to dryness with the aid of MeCN. The residue, which showed positive optical rotation, was identified as glucose by TLC [90] upon comparison with an authentic sample of (+)-D-glucose.

## 4. Results and Discussion

Powdered air-dried flowers were thoroughly defatted by soaking in hexane at room temperature; successively, the biomass was extracted with MeOH, followed by MeOH/ H_2_O (70:30). The yields of the residues B and C resulting from the evaporation of the two extracts were 16.9 and 7.6% (*w*/*w*), respectively. Preliminary phytochemical screening revealed a high content of phenols, phenolic acids, flavonoids, and terpenoids. Two samples of extracts B and C were then filtered through an SPE C-18 cartridge to remove minor amounts of chlorophylls and residual lipophilic components and, successively, through a Sephadex LH-20 column to remove tannins and other polymeric material. The two resulting residues, B’ and C’, were obtained in yields of 95 and 97 %, respectively, from B and C. The total phenolic contents (%, *w*/*w*) of B’ and C’, estimated in terms of gallic acid equivalent by means of the Folin–Ciocalteu reagent [81], were 79.56 (± 1.26)% and 74.91 (± 0.19)%, respectively. Samples of B’ and C’ exhibited no significant antimicrobial activity against the bacteria *Staphylococus aureus*, *Staphylococus mutans*, *Staphylococus pyogenes*, and *Escherichia coli*, and against the fungi *Candida albicans*, *Microsporum canis*, *Aspergillus niger*, and *Bipolaris oryzae*. The antiproliferative activity of residue B’ against MCF-7 breast and A549 lung cancer cells was estimated by the MTS cell viability assay [86]. At the concentration of 600 µg/mL, B’ showed an interesting antiproliferative effects towards the human non-small cell lung cancer A549 cells; notably, the growth inhibition activity decreased only slightly, namely from 48.43% to 44.95%, when the sample concentration was reduced by one tenth to 60 µg/mL. Moving from antiproliferative to antioxidant properties evaluation, the free radical scavenging effects (FRS) of B’ and C’ were tested by the DPPH assay, given that reactive oxygen species (ROS), including several oxygen radicals, are involved in inflammation processes [91]. Significant dose-dependent FRS activities were determined for both B’ and C’, with IC_50_ values of 38.2 and 34.6 mg/mL, respectively. Moreover, at the dose of 62.5 mg/mL, the two residues quenched over 60% DPPH, compared to 59.5% determined for 60 mg/mL of ascorbic acid.

Residue B’ was then separated on a medium pressure liquid chromatographic (MPLC) instrument using a hand-packed reversed-phase column. Elution with a gradient of MeOH in H_2_O led to the isolation of aucubin (**43**, 42.8 mg) and ajugol (**42**, 10.8 mg) in the firstly eluted fractions, whereas mostly a mixture of phenolic derivatives (UV and NMR spectra) was contained in the most polar fractions, which were not analysed further. The structures of the two compounds were determined by spectral analysis. The data (see paragraph 3.1.12) fully corresponded to those reported in the literature [87,88,89].

## 5. Conclusions

Several *Verbascum* species are used in the traditional medicine of Kurdistan. In the first part of this paper, we collected (Table 1) the structures of the main constituents, traditional uses and biological activities reported in the literature for the few *Verbascum* species growing in Kurdistan that have been investigated so far. The most characteristic secondary metabolites are various phenolic derivatives and iridoids and many exhibited interesting bioactivities. Based on these data, we then investigated, for the first time, the contents of polar extracts of *V. calvum* flowers, which are used in Kurdistan for preparing traditional remedies to treat skin burns and inflammations. In standard in vitro assays, a methanol and a hydromethanol extract exhibited weak antimicrobial activities, whereas the methanol extract showed significant antiproliferative effects against an A549 lung cancer cell line. Moreover, both extracts exhibited a significant dose-dependent free radical scavenging action against the DPPH radical, comparable to that of ascorbic acid. In the subsequent MPL separation of the methanol extract, two iridoid glucosides, ajugol (**42**) and aucubin (**43**), were isolated. It is interesting to note that the estimated content of aucubin in dried flowers of *V. calvum* was quite high. In fact, the yield of isolated aucubin was about 0.72% (*w*/*w*); however, the isolation procedure has not been optimized.

Aucubin (**43**) is a low-toxic compound in mice. In addition, an impressive variety of pharmacological effects, including antioxidant, anti-aging, anti-inflammatory, anti-algesic, antifibrotic, anti-tumor, hepatoprotective, neuroprotective and osteoprotective properties, and the promotion of dermal wound healing, have been reported for this compound [92,93,94]. Especially potent are the antioxidant and anti-inflammatory activities determined for aucubin [93]. Due to these biological properties, aucubin is receiving increasing attention by pharmacologists [93]. Concerning the biological properties of ajugol (**42**), a moderate anti-microbial activity against Gram-positive bacteria and *Candida* spp. has been reported [95].

In conclusion, the methanol extract of *V. calvum* flowers has proven to be a rich source of phenolic compounds and iridoid glycosides. The high anti-radical activity of the extract as well as the anti-inflammatory properties reported in the literature for aucubin (**43**), that is one of the main components, validated the use of *V. calvum* in the traditional medicine of Kurdistan as an herbal anti-inflammatory remedy and for topical application to treat skin wounds and burns.

We plan to complete in the future the study of the main compounds isolable from the flowers of *V. calvum*, especially the flavonoid derivatives, and to investigate the contents of leaves and roots extracts, that seem to be different from that of the flowers. Moreover, our attention will be focussed on the preparation of standardized plant extracts and proper pharmaceutical formulations for medicinal applications, as well as on developing an efficient procedure for the quantitative isolation of the valuable bioactive iridoid aucubin (**43**).

## Figures and Tables

**Table 1:** Main constituents, traditional uses and biological activities described for *V*. *cheiranthifolium*, *V*. *songaricum*, *V. speciosum*, and *V. thapsus*.

Species	Traditional Use	Biological Activity	Metabolites
*Verbascum cheiranthifolium* Boiss.	Rheumatism, eczema, earache, menstrual pains, haemorrhoids, oedema, earache and arthralgia [18]	Antioxidant [18], anti-inflammatory [19], anti-ulcerogenic [20], cytotoxic [21], insecticide [22,23], and antimicrobial effects [24].	Aucubin (**43**), catalpol, 6-*O*-(*E*)-coumaroylaucubin, and 6-*O*-[(*E*)-*p*-methoxycinnamoyl]aucubin (**1**) [25]
*Verbascum songaricum* Schrenk	Emmenagogue and to cure infertility [26]	antibacterial [27] and antifungal activities [28]	3-*O*-{[α-L-rhamnopyranosyl-(1→4)-β-D-glucopyranosyl-(1→3)]-[β-D-glucopyranosyl-(1→2)]-β-D-fucopyranosyl}-olea-11,13-diene-3β,23,28-triol (songarosaponin A) (**2**), 3-*O*-{[α-L-rhamnopyranosyl-(1→4)-β-D-glucopyranosyl-(1→3)]-[β-D-glucopyranosyl-(1→2)]-β-D-fuco-pyranosyl}-olea-11-ene-3β,13,23,28-tetrol (songarosaponin B), 3-*O*-{[β-D-glucopyranosyl-(1→4)]-[β-D-glucopyranosyl-(1→3)]-[β-D-glucopyranosyl-(1→2)]-β-D-fucopyranosyl}-13β,28-epoxyolea-11-ene-3β,23-diol (songarosaponin C), 3-*O*-{[β-D-glucopyranosyl-(1→4)]-[β-D-glucopyranosyl-(1→3)]-[β-D-glucopyranosyl-(1→2)]-β-D-fucopyranosyl}-13β,28-epoxyolea-11-ene-3β,16β,23-triol (songarosaponin D), 3-*O*-{[β-D-glucopyranosyl-(l→4)-β-D-glucopyranosyl-(1→3)]-[β-D-glucopyranosyl-(1→2)]-β-D-fucopyranosyl}-olea-11,13-diene-3β,23,28-triol (songarosaponin E), 3-*O*-{[β-D-glucopyranosyl-(1→4)-β-D-glucopyranosyl-(1→3)]-[β-D-glucopyranosyl-(1→2)]-β-D-fucopyranosyl}-olea-11,13-diene-3β,16β,23,28-tetrol (songarosaponin F), and 3-*O*-{[α-L-rhamnopyranosyl-(1→4)-β-D-glucopyranosyl-(1→3)]-[β-D-glucopyranosyl-(1→2)]-β-D-fucopyra- nosyl}-13β,28-epoxyolea-11-ene-3β,16β,23-triol (buddlejasaponin I) [29,30,31]. Poliumoside and verbascoside (**3**) inhibited mammalian DNA polymerases [32]
*Verbascum speciosum* Schrad.	Skin diseases and wound bacterial infection [33]	insecticidal [33], antibacterial [34], antifungal [35] and wound healing potential [36]	Palmitic and oleic acids, (3β,5α)-stigmasta-7,25-dien-3-ol [37]
*Verbascum thapsus* L. [38]	Although mullein has a long history as a favored herbal remedy for the treatment of many disorders [39,40,41,42], high-quality clinical researches have not been conducted so far, and there is no approved drug from this plant [43]. The traditional uses of *V. thapsus* have generally focused on effects aimed at wound healing [44,45,46], and treating Parkinson’s disease [47], diabetes [48,49,50], bronchitis [51], stomachache [52], snake bites [52], spasmodic cough [46], skin diseases [53], asthma [54], and joint pains [54]. The plant is also used as astringent [46] and sedative remedy [55].	antioxidant [49,50,56], antibacterial [39,57,58,59], antiviral [60,61,62,63], anthelmintic [64,65], antihepatitis [66,67], anti-trichomonas [68,69], and anti-leishmanial effects [70].	Luteolin (**4**) [71], apigetrin (apigenin 7-*O*-glucoside) (**5**) [71], 5-*O*-α-L-rhamnopyranoyl(1→3)-[β-D-glucuronopyranosyl-1→6)]-β-D-glucopyranosyl luteolin [72], and the dimer amentoflavone (**6**) [73]. verbathasin A (**7**) [71], ningpogenin (**8**) [71], 10-deoxyeucommiol (**9**) [71], jioglutolide (**10**) [71], 6β-hydroxy-2-oxabicyclo[4.3.0]Δ8-9-nonen-1-one (**11**) [71], (+)-genipin (**12**) [73], α-gardiol (**13**) and β-gardiol (**14**) [73]. Other characteristic terpenoids are buddlindeterpene A-C (**15–17**) [73], and 3α-hydroxy-drimmanyl-8-methanoate [74]. Iridoid glycosides include lateroside (**18**) [73,75,76], harpagoside (**19**) [71,73,75,76], compounds **20–41** [75], ajugol (**42**) [71,73,75,76], aucubin (**43**) [74,76], 6-O-β-xyloxyl aucubin [74], 8-cinnamoylmyoporoside (**44**) [71] and picroside IV (**45**) [73]. Lignan glycosides include compounds **46–50** [77], whereas phenylethanoid glycosides include compounds **51–56** [77], alyssonoside (**57**) [77,78], leucosceptoside B (**58**) [77,78], verbacoside (3) [73,78], leucosceptoside A (**59**) [78], martynoside (**60**) [78], samioside (**61**) [78], and isoverbascoside (**62**) [78]. Sterones and saponins include 24α-methyl-5α-cholestan-3-one, 24-ξ-ethyl-5α-cholestan-22-en-3-one, 24-ξ-ethyl-5β-cholestan-22-en-3-one, 24-ξ-ethyl-5α-cholestan-7-en-3-one, 24α-ethyl-5α-cholestan-Δ7,22-dien-3-one, 24-ξ-ethyl-5α-cholestan-3-one, 24-ξ-ethyl-5β-cholestan-3-one [74], and 3-O-fucopyranosylsaikogenin F (**63**) [71].Verbascoside (**3**) exhibited anti-inflammatory properties [79], whereas luteolin (**4**) and saponin 63 induced apoptosis of A549 lung cancer cells [71].

**Table 2:** Minimum inhibition concentrations (MICs) of B’ and C’ from flowers of *Verbascum calvum*.

Sample.	*Candida albicans*	*Aspergillus niger*	*Microsporum canis*	*Bipolaris oryzae*
Extract B’	>5 mg/mL	>5 mg/mL	1 mg/mL	>1 mg/mL
Extract C’	>5 mg/mL	>5 mg/mL	-	>1 mg/mL

**Table 3:** Percentages of tumor cell growth inhibition by sample B’.

Cell Line	600 µg/mL	60 µg/mL	6 µg/mL	0.6 µg/mL	0.06 µg/mL
A549	48.43	44.95	14.69	–	–
MCF-7	19.62	18.35	–	–	–

**Table 4:** ^1^H-NMR (300 MHz, MeOH-*d*_4_, TMS; δ_H_ in ppm, *J* in Hz) and ^13^C-NMR (75 MHz, MeOH-*d*_4_, TMS, δ_C_ in ppm) spectral data of aucubin (**43**) and ajugol (**42**) isolated from flowers of *Verbascum calvum.*

Proton/Carbon	Aucubin (43)		Ajugol (42)	
	^1^H *^a^*	^13^C *^b^*	^1^H *^a^*	^13^C *^b^*
1	1H, 4.96 d (7.0)	97.7 (CH)	1H, 5.47 d (2.2)	93.7 (CH)
3	1H, 6.31 dd (6.0 and 1.8)	141.6 (CH)	1H, 6.16 dd (6.3 and 1.9)	140.4 (CH)
4	1H, 5.10 dd (6.0 and 4.0)	105.7 (CH)	1H, 4.78–4.82 (m)	105.9 (CH)
5	1H, 2.60–2.70 (m)	46.3 (CH)	1H, 2.69–2.74 (m)	41.2 (CH)
6	1H, 4.39–4.48 (m)	82.8 (CH)	1H, 3.90 ddd (5.6, 5.0, and 3.0)	78.0 (CH)
7	1H, 5.76 br s (t)	130.3 (CH)	1H, 1.79 dd (13.5, 5.0);1H, 2.05 dd (13.5 and 5.6)	50.0 (CH_2_)
8	-	148.0 (C)	-	79.5 (C)
9	1H, 2.89 br t (7.2)	47.9 (CH)	1H, 2.53 dd (9.5 and 2.2)	51.8 (CH)
10	2H, ABq centered at 4.25 (15.4)	61.4 (CH_2_)	3H, 1.30 s	25.2 (CH_3_)
1′	1H, 4.68 d (7.8)	99.9 (CH)	1H, 4.63 d (7.9)	99.4 (CH)
2′	1H, 3.21 dd (7.8 and 9.0)	74.9 (CH)	1H, 3.21 dd (8.0 and 9.0)	74.8 (CH)
3′	1H, 3.34 t (9.0)	77.9 (CH)	1H, 3.30–3.37 m	77.8 (CH)
4′	1H, 3.28 t (9.0)	71.6 (CH)	1H, 3.20–3.25 m	71.7 (CH)
5′	1H, 3.28–3.33 m	78.3 (CH)	1H, 3.28–3.33 m	78.2 (CH)
6′	1H, 3.65 dd (12.2 and 5.2);1H, 3.87 dd (12.2 and 1.5)	62.7 (CH_2_)	1H, 3.66 dd (12.0 and 5.2);1H, 3.91 dd (12.0 and 1.5)	62.9 (CH_2_)

*^a^* Proton assignments are based on COSY and HSQC experiments. *^b^* The number of hydrogens attached to each carbon was determined by DEPT experiments. Carbon assignments were established by HSQC and HMBC experiments.

## Supplementary Materials

NMR spectra of aucubin and ajugol, as well as bar-graphs of the antiproliferative activity of extract B’, are available online at https://www.mdpi.com/2223-7747/9/9/1066/s1.
