# Supplementary material for: Phytochemistry of Verbascum Species Growing in Iraqi Kurdistan and Bioactive Iridoids from the Flowers of Verbascum calvum
Source: Plants (Basel). 2020 Aug 20;9(9):1066. doi: 10.3390/plants9091066 (PMC7569995; doi:10.3390/plants9091066)
Supplement: Supplementary file 1 [file plants-09-01066-s001.pdf]

SUPPLEMENTARY INFORMATION

Phytochemistry of *Verbascum* species growing in Iraqi Kurdistan and Bioactive Iridoids from the Flowers of *Verbascum calvum*

Hawraz Ibrahim M. Amin, Faiq H. S. Hussain, Gianluca Gilardoni, Zaw Min Thu, Marco Clericuzio, Giovanni Vidari

NMR spectra of aucubin (**43**)

Figure 1S. ^{1}H-NMR spectrum (200 MHz) of aucubin (in CD_3OD) page 2.
Figure 2S. ^{1}H-NMR spectrum (300 MHz) of aucubin (in CD_3OD) page 3.
Figure 3S. ^{13}C-NMR spectrum (75 MHz) of aucubin (in CD_3OD) page 4
Figure 4S. LC-MS of aucubin in MeOH page 5

NMR spectra of ajugol (**42**)

Figure 5S. ^{1}H-NMR spectrum (200 MHz) of ajugol (in CD_3OD) page 6.
Figure 6S. ^{1}H-NMR spectrum (300 MHz) of ajugol (in CD_3OD) page 7.
Figure 7S. ^{13}C-NMR spectrum (75 MHz) of ajugol in (CD_3OD) page 8
Figure 8S. DEPT spectrum (75 MHz) of ajugol in (CD_3OD) page 9
Figure 9S. COSY spectrum (300 MHz) of ajugol (in CD_3OD) page 10
Figure 10S. LC-MS of ajugol in methanol page 11

Tumor cell viability test (MTS assay) of residues B'

Figure 11S. Bar-graphs of the antiproliferative activity of extract B' against A549 and MCF-7 tumor cell lines (MTS assay) page 12

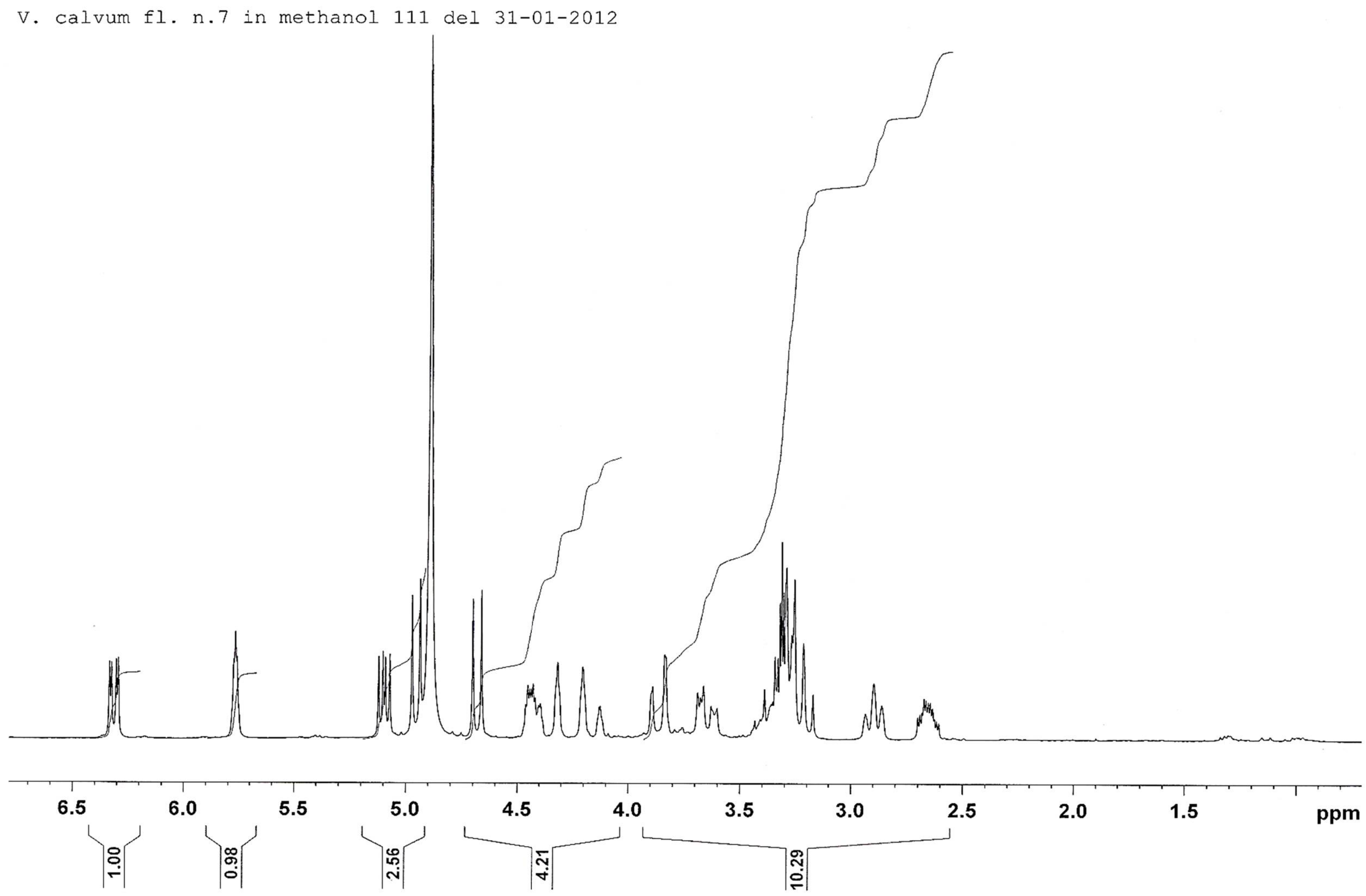

Figure 1S. ^{1}H-NMR spectrum (200 MHz) of aucubin (in CD_3OD).

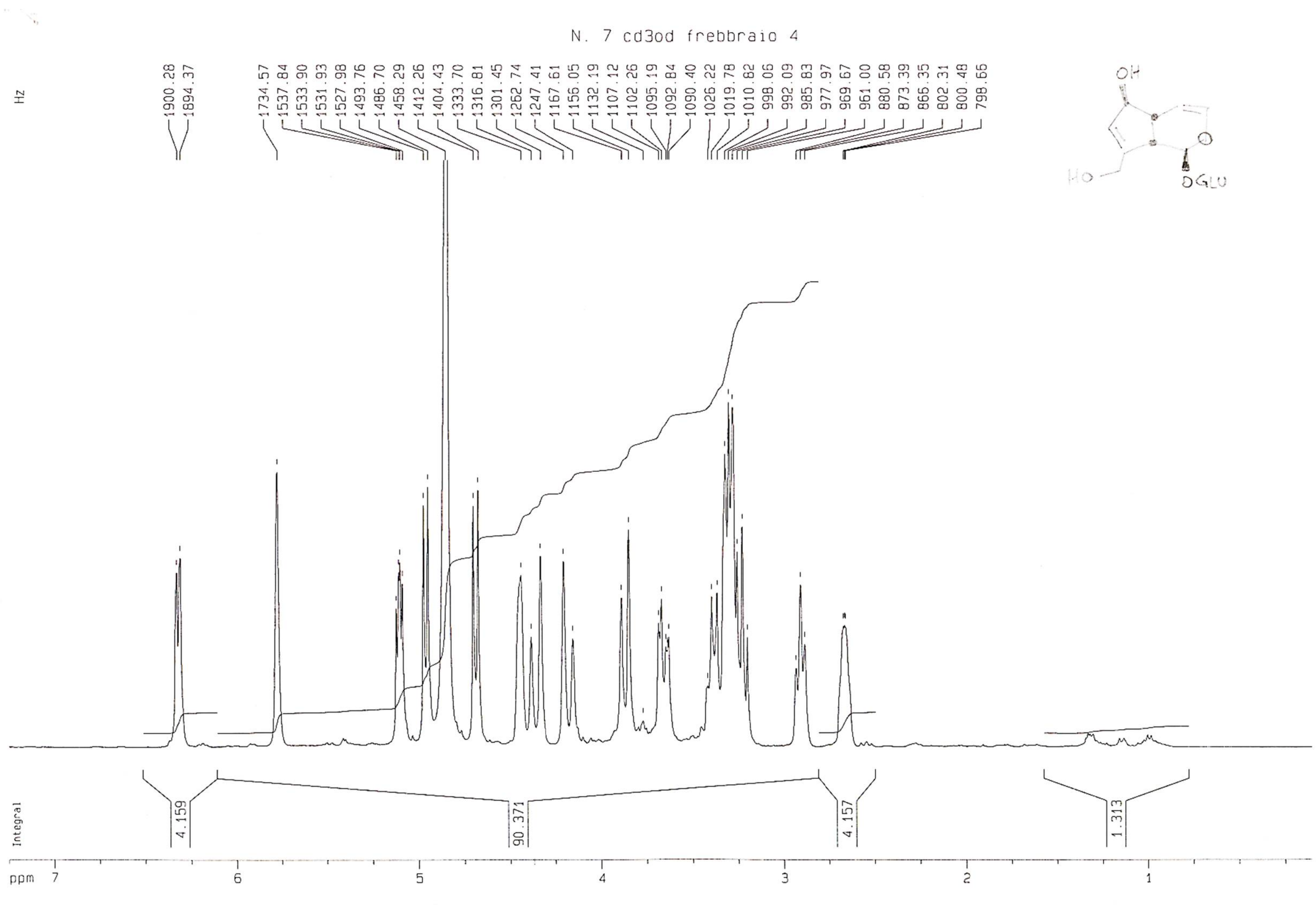

Figure 2S. ^{1}H-NMR spectrum (300 MHz) of aucubin (in CD_3OD).

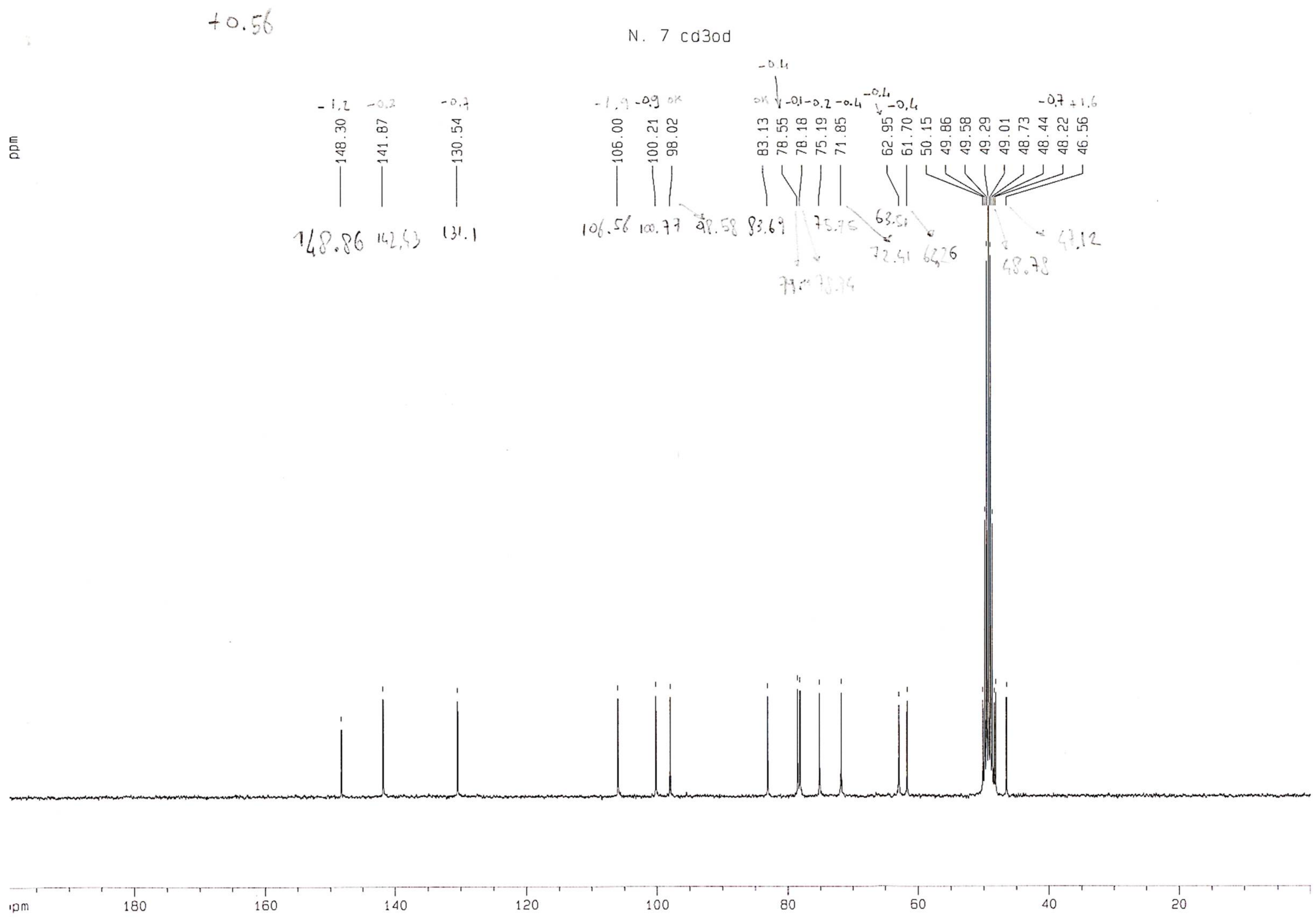

Figure 3S. ^{13}C-NMR spectrum (75 MHz) of aucubin (in CD_3OD).

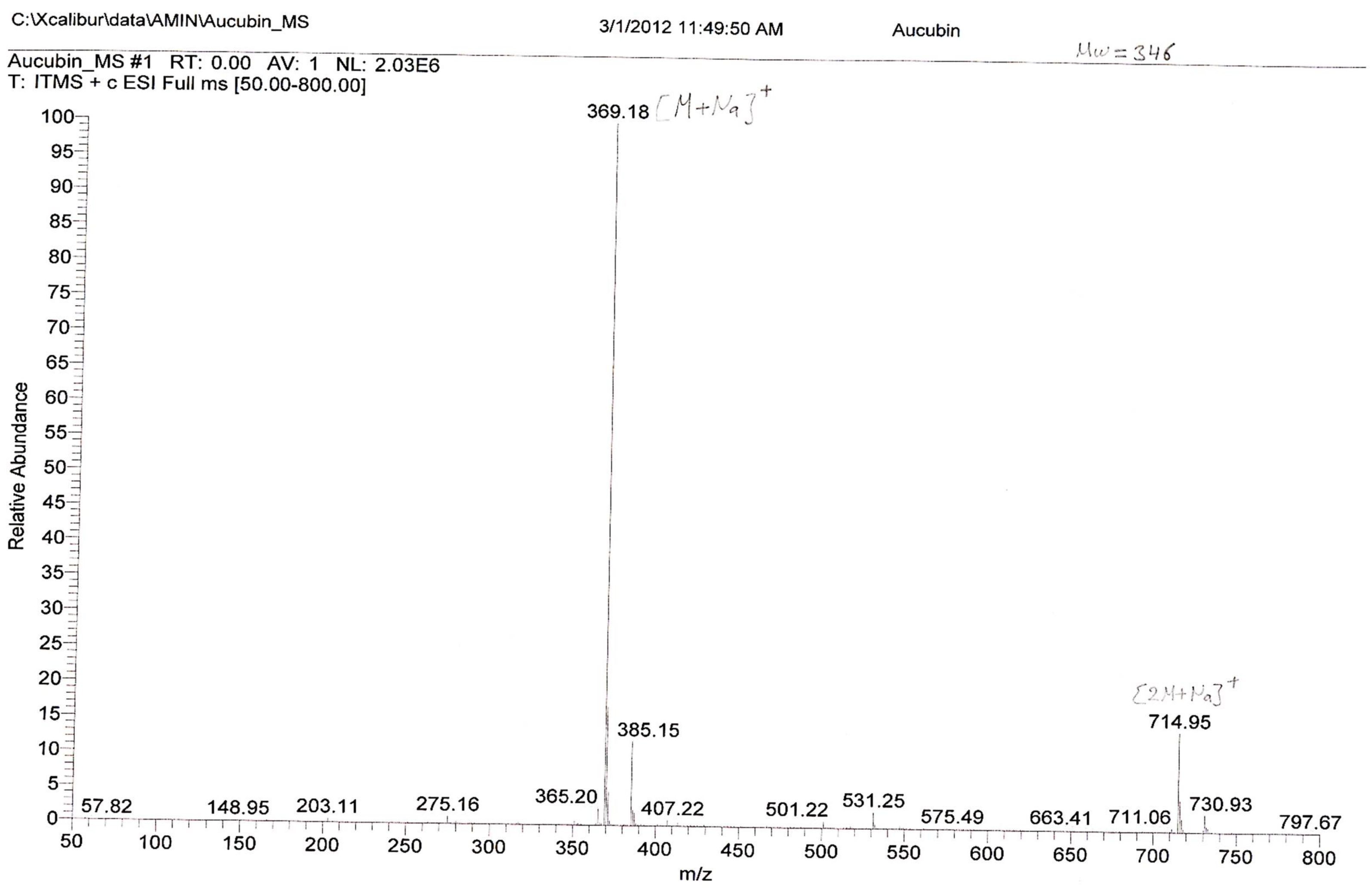

Figure 4S. LC-MS of aucubin in methanol.

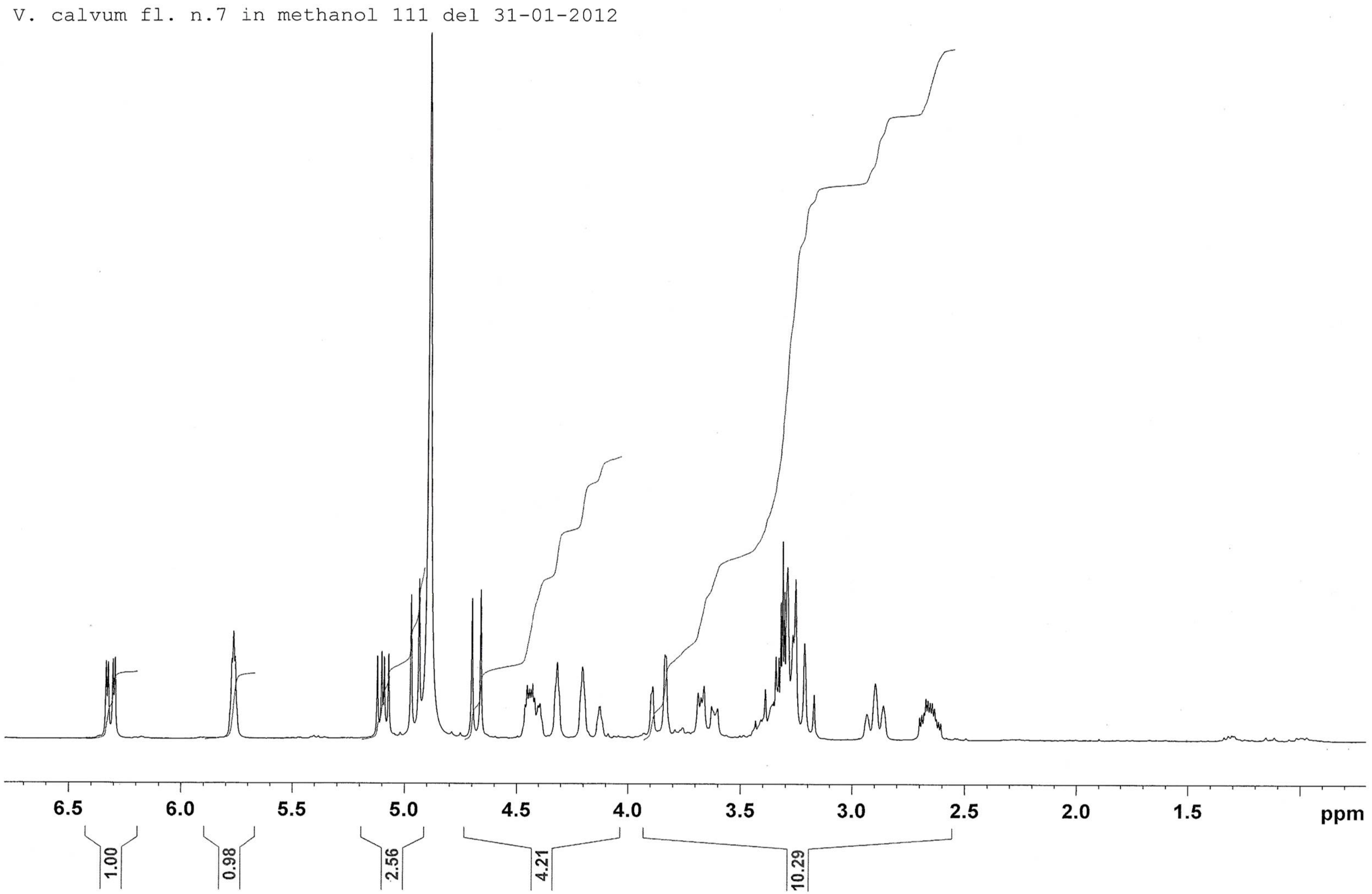

Figure 5S. ^{1}H-NMR spectrum (200 MHz) of ajugol (in CD_3OD).

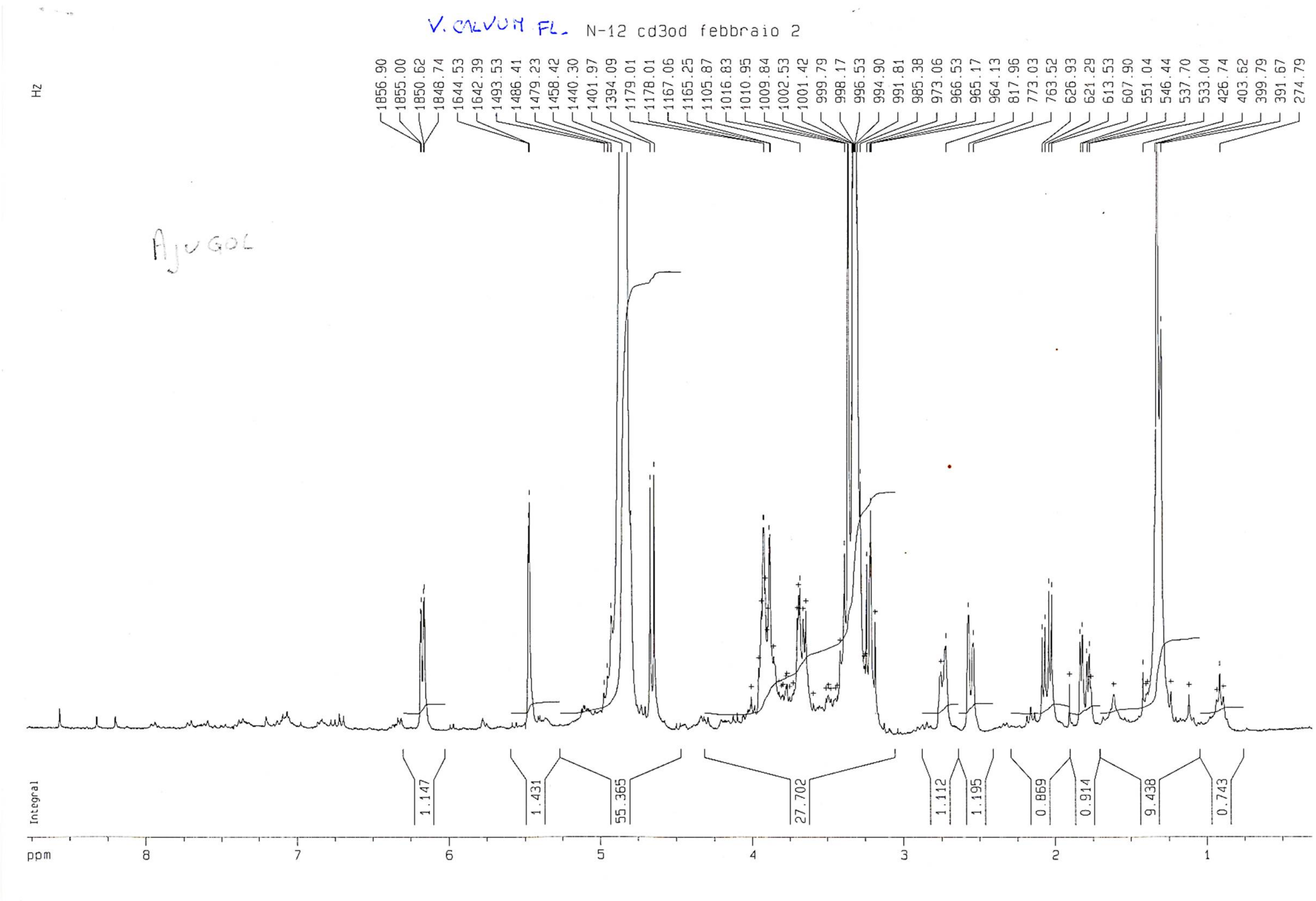

Figure 6S. ^{1}H-NMR spectrum (300 MHz) of ajugol (in CD_3OD).

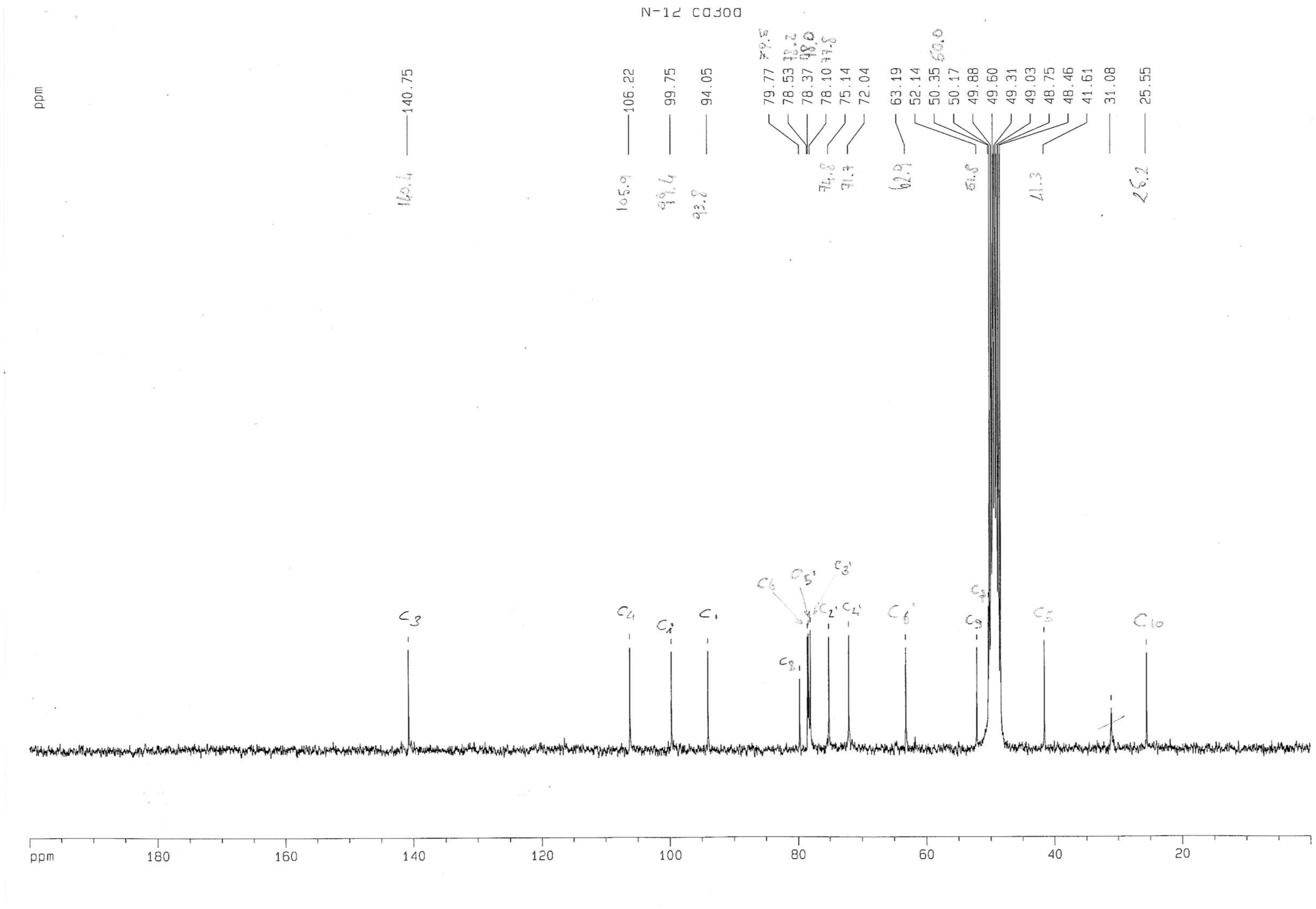

Figure 7S. ^{13}C-NMR spectrum (75 MHz) of ajugol in (CD_3OD).

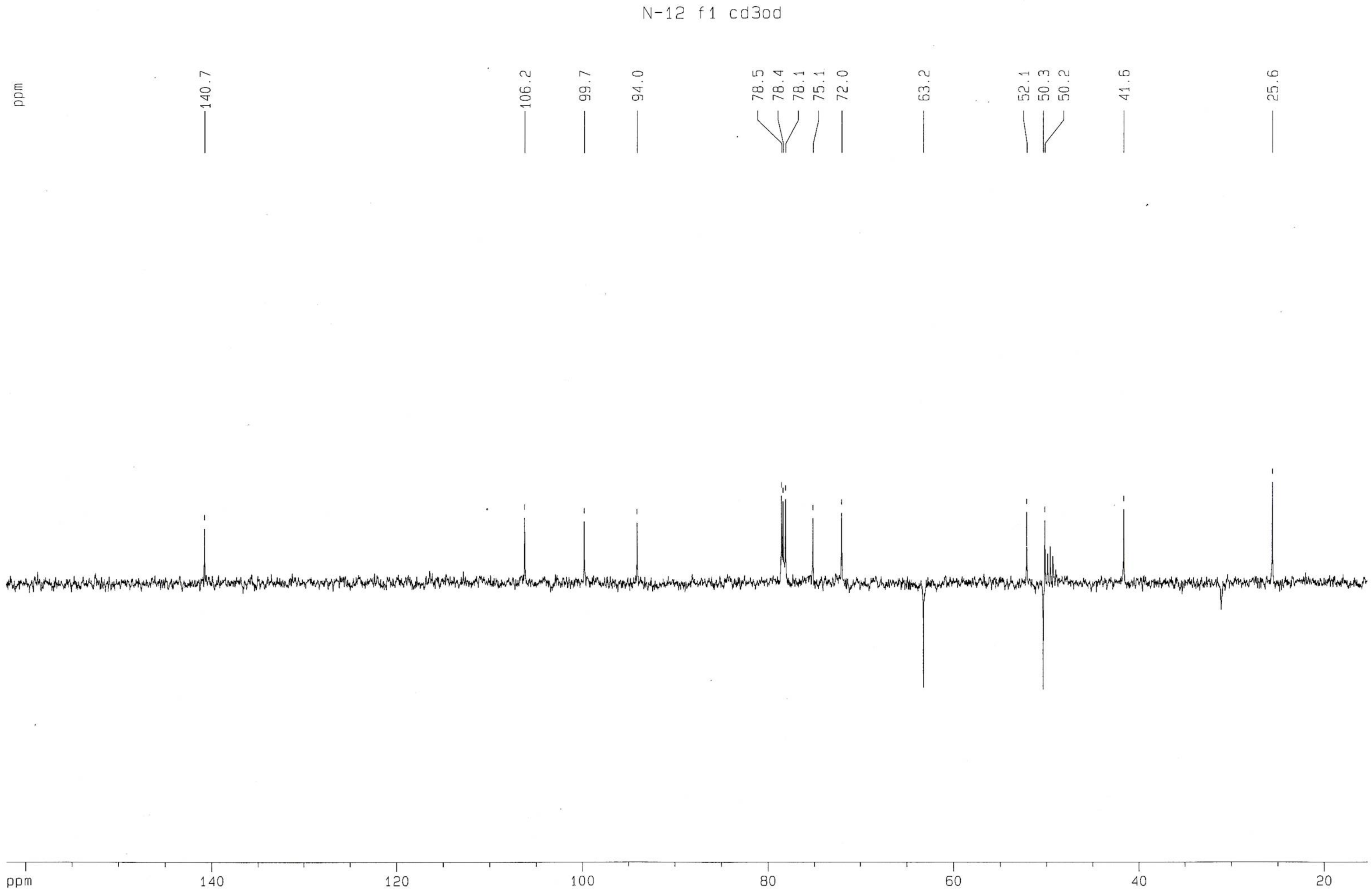

Figure 8S. DEPT spectrum (75 MHz) of ajugol in (CD_3OD).

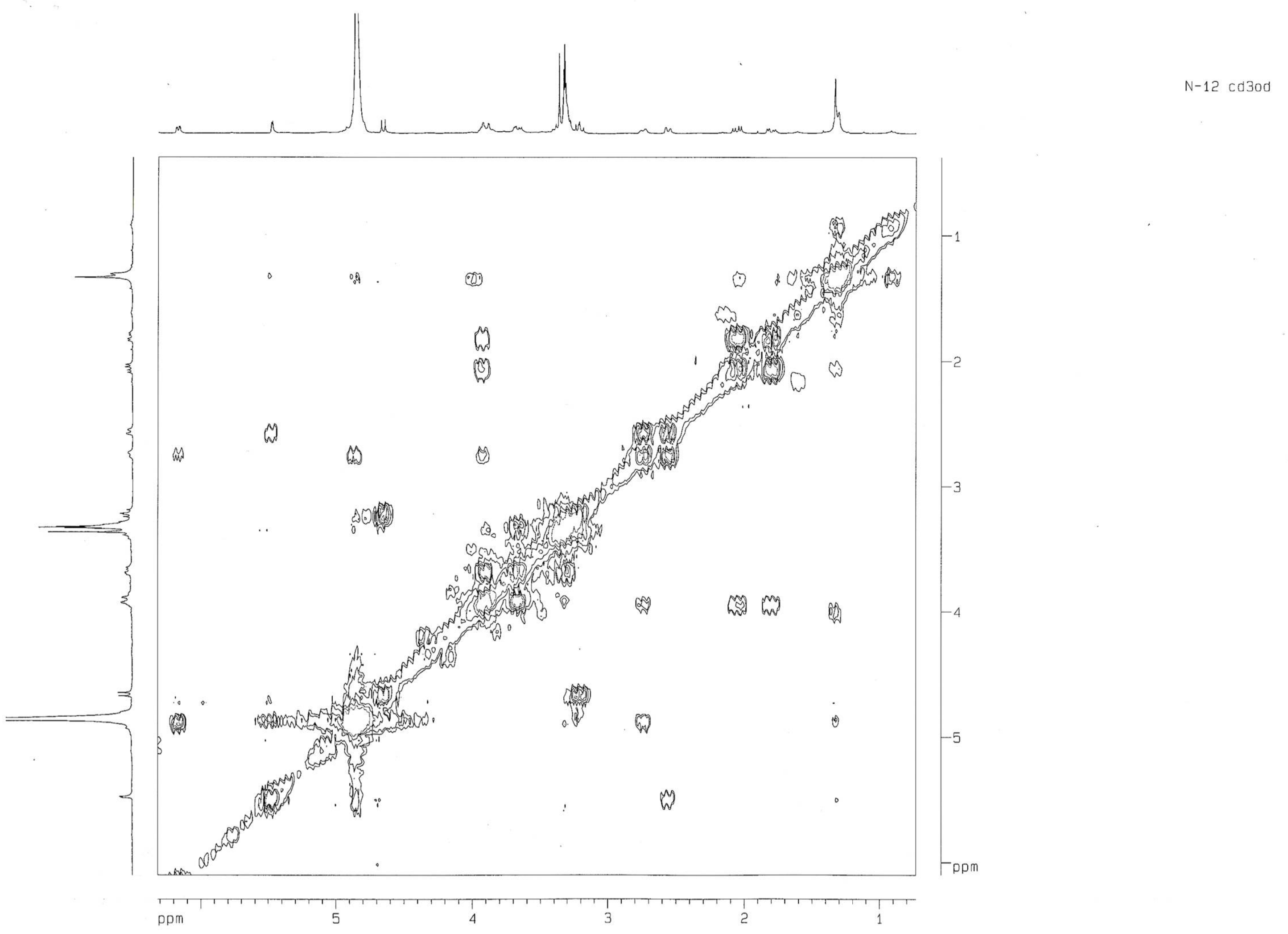

Figure 9S. COSY spectrum (300 MHz) of ajugol (in CD_3OD).

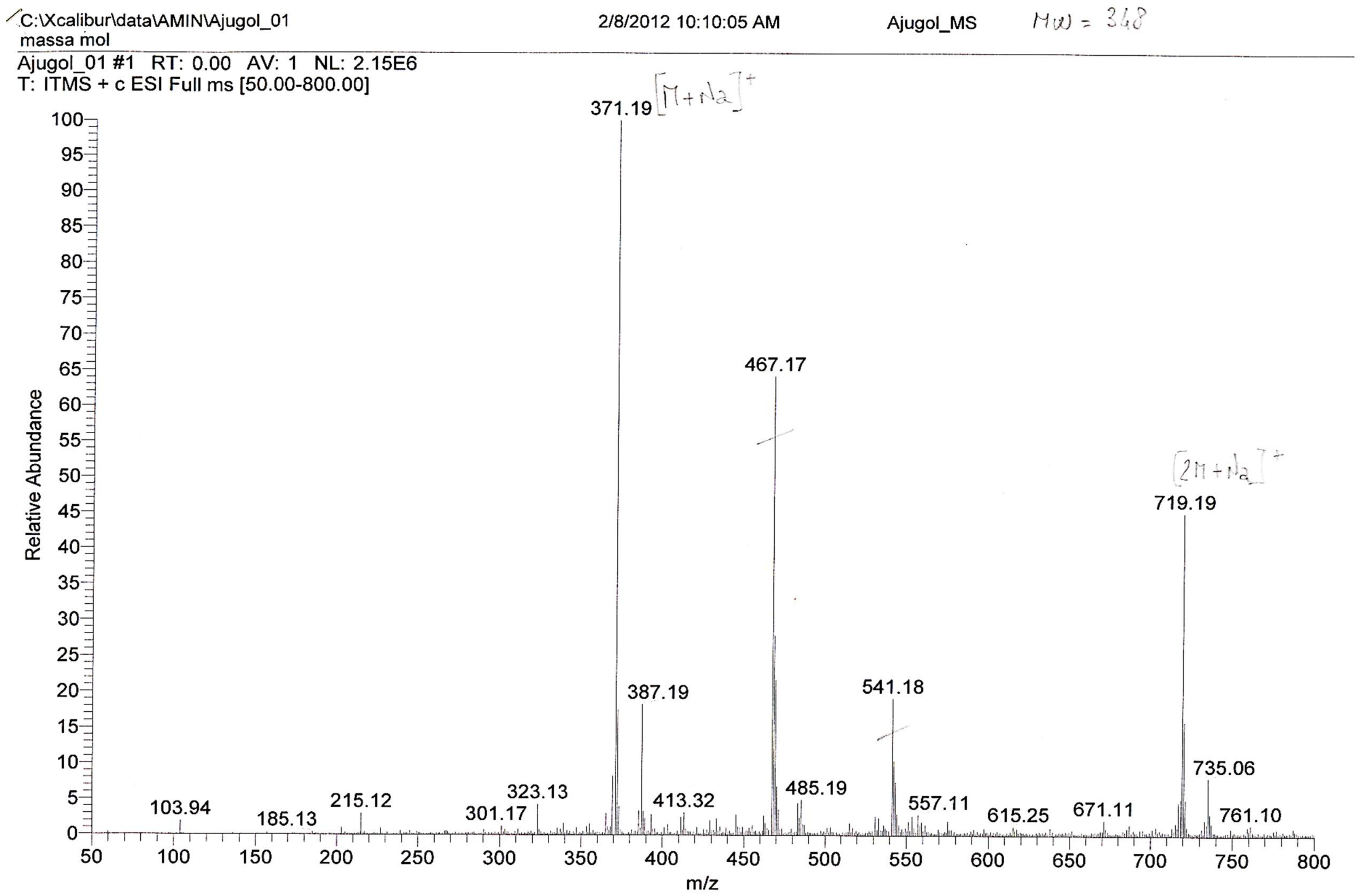

Figure 10S. LC-MS of ajugol in methanol.

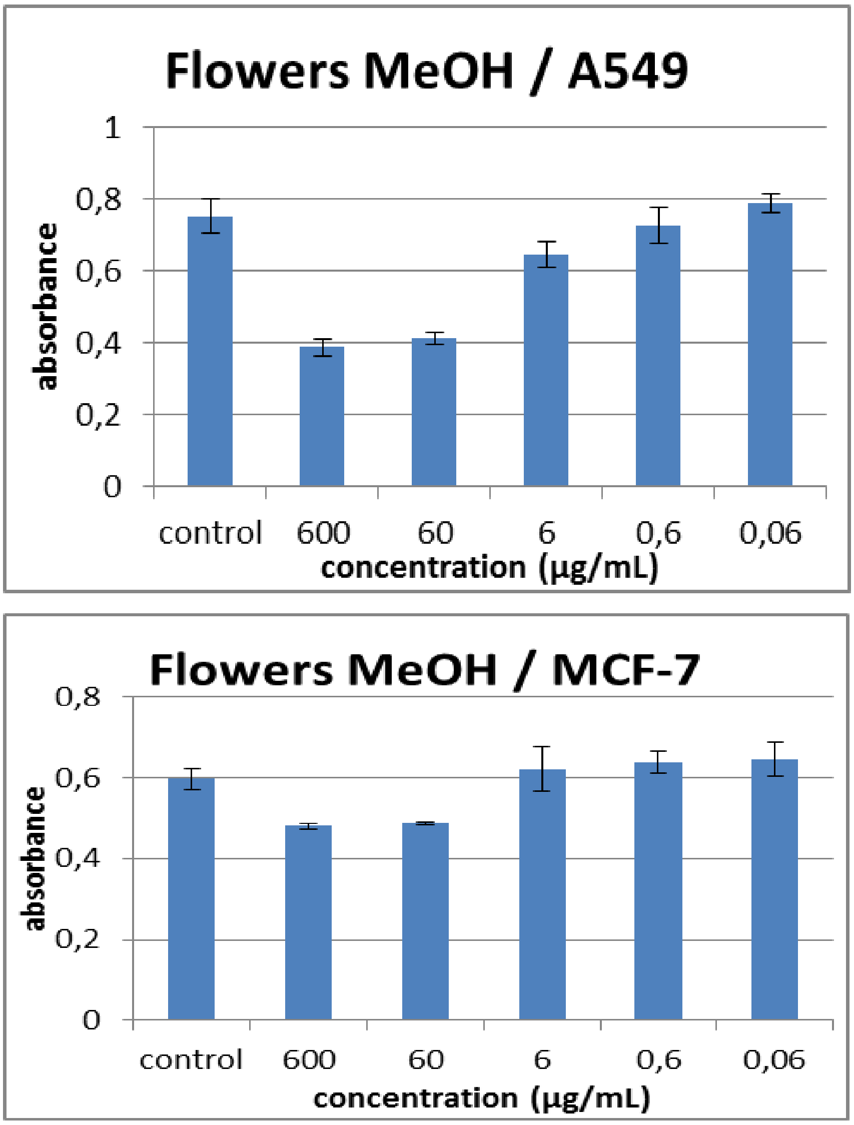

Figure 11S. Antiproliferative activity of extract B' against A549 and MCF-7 tumor cell lines (MTS assay). The percentages of growth inhibition of tumor cells (treated with different concentration of extract B'), compared to the control, are calculated from the absorbances at 490 nm.
